# Hirudin Regulates Vascular Function in Chronic Renal Failure through Modulating Macrophage Polarization

**DOI:** 10.1155/2022/6043698

**Published:** 2022-04-19

**Authors:** Bo Chen, Xunfang Ding, Yanbo Yang

**Affiliations:** Department of Nephrology, Lianyungang Hospital of Traditional Chinese Medicine, Lianyungang, Jiangsu 222000, China

## Abstract

Excessive inflammation is responsible for arteriovenous fistula (AVF) failure, which determines the therapeutic effect of chronic renal failure (CRF). Macrophage polarization is of great significance in the inflammatory response. Hirudin (Hiru) has been reported to possess a definite anti-inflammatory effect. This study is to uncover the impacts of Hiru on classically (M1)/alternatively (M2) macrophage polarization in the CRF rat model and rat vascular smooth muscle cells (VSMCs). After the CRF rat model was administrated with different concentrations of Hiru, blood urea nitrogen (BUN) and serum creatinine (Scr) levels were tested. H&E staining was to detect vascular injury, and IHC assay was to analyze inducible nitric oxide synthase (iNOS) and arginase-1 (Arg-1) expressions in vascular tissues. Levels of inflammatory factors were examined by ELISA. Besides, western blot was to estimate the levels of marker proteins related to macrophage, proliferation, and apoptosis. CCK-8 was to measure cell viability. We discovered that Hiru alleviated renal function injury and vascular injury, exacerbated VSMC hyperplasia, and stimulated the differentiation and activation of M1 macrophage towards M2 macrophage in vivo. Moreover, after treatment with lipopolysaccharide (LPS)/IFN-gamma (IFN-*γ*), the increased M1/M2 ratio and enhanced levels of inflammatory factors were observed. Furthermore, Hiru boosted the proliferation and ameliorated the inflammatory response and apoptosis of rat VSMCs during the process of coincubation of M1-conditioned medium (CM). Collectively, Hiru played a protective role against vascular injury in CRF directly or through its influence on M1 macrophage polarization and inflammation.

## 1. Introduction

Chronic kidney disease (CKD) is a primary public health issue with an association of expensive costs, mortality, and morbidity [1]. CKD is commonly classified into five stages based on the glomerular filtration rate (GFR): Stage 1 (eGFR > 90 + ACR > 30), Stage 2 (eGFR 60‐89 + ACR > 30), Stage 3 (eGFR 30‐59), Stage 4 (eGFR 29‐15), and Stage 5 (eGFR < 15) [2]. Stage 5 CKD, also known as end-stage kidney disease, is mainly defined as chronic renal failure (CRF). Dialysis and transplantation are acknowledged as major therapies for CRF patients [3]. Of these, hemodialysis (HD) is a widely accepted form of dialysis, which achieves blood purification by removing waste products, harmful electrolytes, and free water from the blood [4]. Arteriovenous fistula (AVF) is the preferred vascular access during HD process, which provides sufficient blood to ensure the adequacy of HD treatment [5]. The AVF maturation phase is involved in vascular proliferation, and dilated remodeling (mainly smooth muscle cells) is required [6]. However, AVF maturation failure remains a major obstacle for the effectiveness of HD treatment [7]. Hence, the exploration of promising drugs in smooth muscle cell dysfunction may be beneficial to the successful maturation of AVF in HD, so as to improve the survival rate of CRF patients.

Macrophages are a type of white blood cell present in all body parts and deemed as vital players in development, homeostasis, tissue repair, and immunity [8, 9]. Under different environmental factors, macrophages can be transformed into a variety of functional phenotypes, of which two distinct subsets are pro- (M1) or anti-inflammatory (M2) macrophages [10]. As reported, M1/M2 macrophage homeostasis is involved in the renal microenvironment and may participate in the progression of CKD [11]. More importantly, macrophages are related to AVF wall thickening and AVF maturation [12]. Consequently, it is of great significance for AVF maturation to abrogate the activation and differentiation of M1 macrophages or to accelerate the transformation of M1 macrophages towards M2 macrophages.

Hirudin (Hiru), a peptide naturally occurring in leeches and their salivary glands, possesses anticoagulative property [13]. Also, more attention has been paid on the anti-inflammatory effects of Hiru. A large number of researches have mentioned that Hiru decreases inflammatory response in renal interstitial fibrosis [14], diabetic nephropathy [15], and acute cardiovascular disease [16]. Meanwhile, Pang et al. have uncovered that Hiru could elevate the expression of arginase-1 (Arg-1), a functional marker of M2 phenotype in high glucose-induced diabetes [17]. Accordingly, we made a conjecture that Hiru may regulate M1 and M2 macrophage polarization phenotype in CRF.

This paper concentrated on clarifying the function of Hiru on M1/M2 macrophage polarization in CRF rat models and in rat vascular smooth muscle cells (VSMCs) via simulating the vascular physiology changes during AVF.

## 2. Materials and Methods

### 2.1. Animal Model

A total of 30 eight-week-old male Wistar rats (200-220 g) were purchased from Shanghai SLAC Laboratory Animal Company Limited (Shanghai, China). Rats in the CRF group were given 250 mg/kg adenine (Sigma-Aldrich, St. Louis, MO) by oral gavage once daily for 14 days, and these rats were administrated with adenine every other day for the next 14 days. The sham group was given the same amount of normal saline. For the Hiru-treated group, rats were intraperitoneally injected with different doses of Hiru (10, 20, and 40 IU/kg), respectively [18]. Subsequently, rats were euthanized at Day 0 (D0), D7, D14, and D28, respectively. The blood samples and arterial tissues were collected for subsequent experiments. The animal study was approved by the Animal Research Ethics Committee of Lianyungang Hospital of Traditional Chinese Medicine.

### 2.2. Measurement of Blood Urea Nitrogen (BUN) and Serum Creatinine (Scr)

After centrifugation at 3,000 rpm for 20 min at 4°C, blood samples were separated to obtain serum. The levels of renal function parameters BUN and Scr were detected by commercial kits from Nanjing Jiancheng Bioengineering Institute (Nanjing, China).

### 2.3. Hematoxylin and Eosin (H&E) Staining

To mimic external vascular injury, the blood vessels were compressed and then loosened in the sham group. Rat aortic tissues were immersed in 10% formalin, followed by embedding and paraffin sectioning. Then, hematoxylin solution (H8070; Solarbio) and eosin (Solarbio Life Sciences) were applied to stain the slices. A light microscope (Leica Microsystems, Wetzlar, Germany) was utilized to capture the images.

### 2.4. Immunocytochemistry (IHC)

After being cut into sections (5 *μ*m), rat vascular tissues were labeled with antibodies specific for iNOS (Abcam, 1 : 2000, ab283655) and Arg-1 (Cell Signaling Technology, 1 : 50, #93668), followed by treatment of an immunohistochemical labeling kit (MaxVision Biotechnology, Fuzhou, China). At last, iNOS and Arg-1 expressions were detected under a light microscope (Leica Microsystems, Wetzlar, Germany).

### 2.5. Enzyme-Linked Immunosorbent Assay (ELISA)

With the aid of ELISA Kits from Abcam, the levels of tumor necrosis factor alpha (TNF-*α*; ab236712), interleukin 6 (IL-6; ab234570), and interleukin 10 (IL-10; ab214566) were estimated in accordance with the manufacturer's instructions. The OD value was read at 450 nm using a microplate reader (Molecular Devices, USA).

### 2.6. Cell Culture

RAW264.7 cells were provided by the Cell Bank of the Chinese Academy. Primary rat VSMCs were obtained and isolated from rat thoracic aortas [19]. All cells were grown in Dulbecco's modified Eagle's medium (DMEM, Corning) that was inclusive of 10% fetal bovine serum (FBS; Gibco) and 1% antibiotics and kept in culture at 37°C with 5% CO_2_. VSMCs between passages 3 and 8 were used for the following experiments. To induce M1 polarization, RAW264.7 cells were stimulated by IFN-*γ* and LPS for 24 h and then washed with fresh medium for three times and continued to incubate for 24 h. The supernatant was harvested and filtered through a 0.22 *μ*m filter to remove cell debris [20, 21]. The M1-conditioned medium (CM) was obtained. Also, Hiru at concentrations of 5, 10, and 20 U/ml supplemented with M1-CM was to treat VSMCs [22]. Besides, Hiru (5, 10, and 20 U/ml concentrations) was added during the induction and culture of M1 macrophages and the medium was replaced at the end of the process. Finally, the medium was collected, respectively, after further incubation of the macrophages to coculture with VSMCs.

### 2.7. Cell Counting Kit-8 (CCK-8) Assay

VSMCs (5,000 cells/well) were seeded into 96-well plates and maintained for 12, 48, or 72 h. After addition of 10 *μ*l CCK-8 solution (Dojindo Laboratories, Kumamoto, Japan) at 37°C for 2 h, cell viability was observed by absorbance at 450 nm with a microplate reader (Molecular Devices, USA).

### 2.8. Western Blot

Cell lysis buffer (Promega, Madison, WI) was employed for the dissolution of total protein, and 10% SDS-PAGE was used for the separation of protein samples. Afterwards, proteins were loaded onto PVDF membranes (Millipore, Bedford, MA) and impeded with 5% nonfat milk at 37°C for 2 h. The primary antibodies specific for CD86 (Abcam, 1 *μ*g/ml, ab112490), iNOS (Abcam, 1 : 1000, ab283655), CD206 (Cell Signaling Technology, 1 : 1,000, #24595), Arg-1 (Cell Signaling Technology, 1 : 1000, #93668), Bcl-2-associated X (Bax; Abcam, 1 : 1000, ab32503), B-cell lymphoma 2 (Bcl-2; Abcam, 1 : 1000, ab194583), cleaved caspase-3 (Cell Signaling Technology, 1 : 1000, #9664), caspase-3 (Abcam, 1 : 2000, ab184787), matrix metallopeptidase 2 (MMP2; Abcam, 1 : 1000, ab92536), alpha-smooth muscle actin (*α*-SMA; Cell Signaling Technology, 1 : 1000, #19245), and glyceraldehyde-3-phosphate dehydrogenase (GAPDH; Abcam, 1 : 10000, ab181602) were added in the membranes for overnight incubation at 4°C. After incubation with HRP-conjugated secondary antibody (Abcam, 1 : 1000, ab133470) for 1 h at room temperature, protein blots were visualized using an ECL kit (Millipore, Bedford, MA, USA).

### 2.9. Statistical Analysis

All experiments were repeated independently in triplicate. Data were displayed as the mean ± standard deviation. Student's *t*-test was applied to assess the differences when two groups were compared. Differences between multiple groups were compared with the aid of one-way ANOVA, followed by Tukey's post hoc test. All analyses were performed with the use of GraphPad Prism 8.0 (La Jolla, CA, USA). *P* < 0.05 was accepted as statistically significant.

## 3. Results

### 3.1. Hiru Attenuates Renal Injury in a Dose- and Time-Dependent Manner and Regulates M1/M2 Macrophages in CRF Rat Models

**Figure 1:**
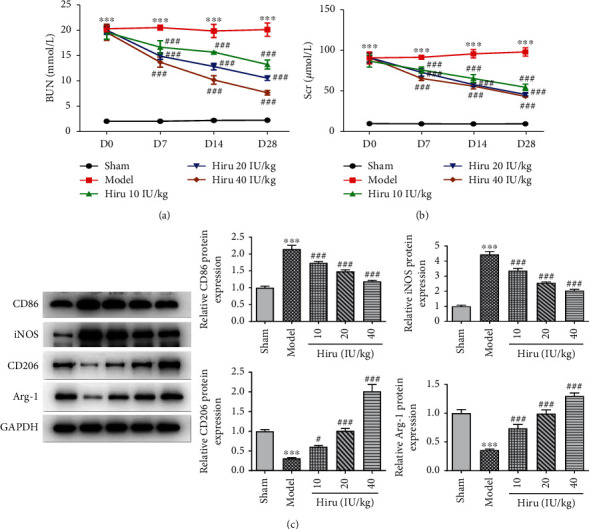
Hiru attenuates renal injury in a dose- and time-dependent manner and regulates M1/M2 macrophages in CRF rat models. The commercially available kits were to test (a) BUN and (b) Scr levels. (c) Western blot was to analyze the protein levels of CD86, iNOS, CD206, and Arg-1. ^∗∗∗^*P* < 0.001 vs. sham. ^#^*P* < 0.05 and ^###^*P* < 0.001 vs. model.

To figure out the functions of Hiru on renal function, the levels of renal injury markers BUN and Scr were detected. It was clearly observed that BUN and Scr levels were distinctly enhanced in CRF rat models relative to the sham group. Moreover, Hiru notably led to the reduction in BUN and Scr levels in a dose-dependent manner at D7, D14, and D28 (Figures 1(a) and 1(b)). Intriguingly, as compared to the sham group, the protein levels of M1 markers (CD86 and iNOS) in the model group were remarkably elevated, while M2 markers (CD206 and Arg-1) were markedly declined in the model group. After administration of increasing concentrations of Hiru, CD86 and iNOS expressions were decreased whereas CD206 and Arg-1 expressions were increased (Figure 1(c)), which implied that Hiru induced M2 polarization in CRF rat models. To be summarized, Hiru mitigated renal injury, suppressed M1, and stimulated M2 polarization.

### 3.2. Hiru Modulates Vascular Injury and Macrophage M1/M2 Polarization in CRF Rat Models

**Figure 2:**
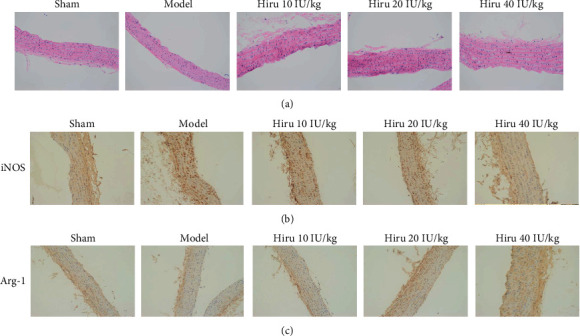
Hiru modulates vascular injury and macrophage M1/M2 polarization in CRF rat models. (a) The endothelial injury and vascular smooth muscle hyperplasia were measured by H&E staining. IHC assay was to detect (b) iNOS and (c) Arg-1 expression in rat vascular tissues. Scale bar, 50 *μ*m.

The changes in endothelial injury and smooth muscle layers were observed through H&E staining. The experimental results manifested that the aggravated endothelial injury and the attenuated vascular smooth muscle hyperplasia in the model group were reversed by Hiru in a dose-dependent manner (Figure 2(a)). Also, as depicted in Figures 2(b) and 2(c), in comparison with the model group, a decline in iNOS expression and an elevation in Arg-1 expression were observed in the Hiru groups (10, 20, and 40 IU/kg). In a word, Hiru alleviated vascular injury while accelerated M2 polarization in CRF rat models.

### 3.3. M1 Macrophage Enhances Inflammatory Response

**Figure 3:**
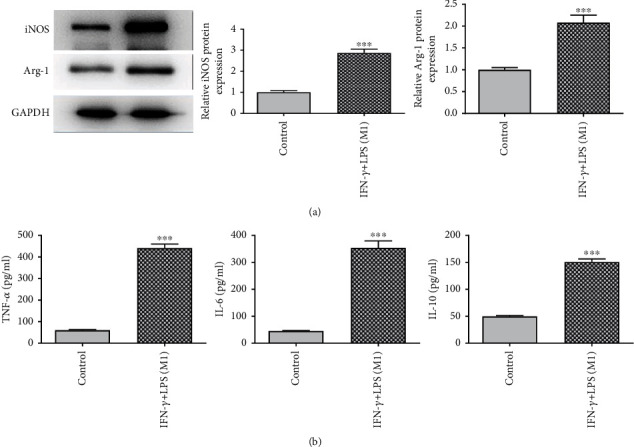
M1 macrophage enhances inflammatory response. (a) Western blot was to analyze the protein levels of iNOS and Arg-1. (b) ELISA assay was to examine the activities of TNF-*α*, IL-6, and IL-10. ^∗∗∗^*P* < 0.001 vs. control.

To obtain the M1 phenotype, RAW264.7 cells were induced by IFN-*γ* and LPS for 24 h. Then, the protein levels of iNOS and Arg-1 were detected by western blot. It was discovered that the iNOS protein level was obviously enhanced while the Arg-1 protein level was greatly lessened in the presence of IFN-*γ* and LPS (Figure 3(a)). More importantly, the levels of inflammatory factors TNF-*α*, IL-6, and IL-10 were dramatically increased in RAW264.7 cells treated with IFN-*γ* and LPS (Figure 3(b)). In summary, M1-polarized macrophages resulted in inflammation.

### 3.4. Hiru Serves as a Suppressor in Macrophage towards M1 Activation and VSMC Apoptosis While Functions as a Promoter in VSMC Proliferation

**Figure 4:**
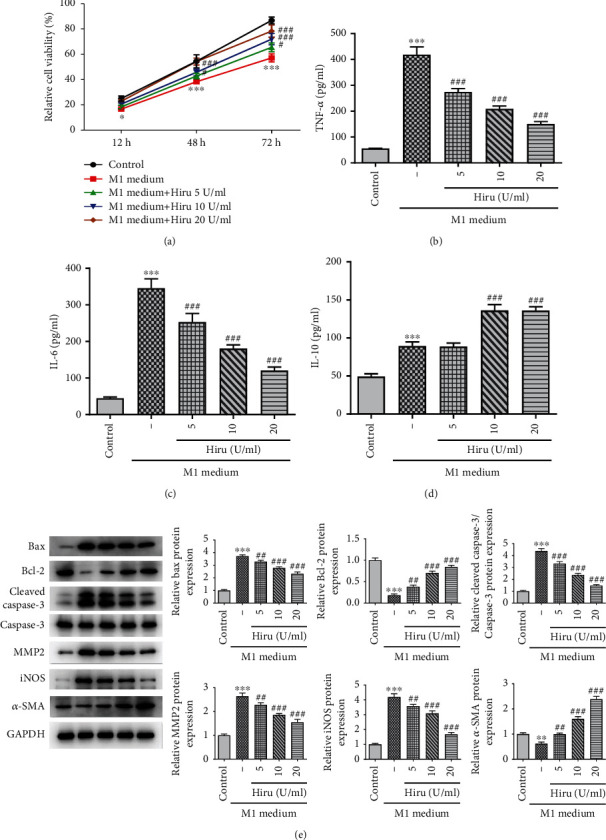
Hiru serves as a suppressor in macrophage towards M1 activation and VSMC apoptosis while functions as a promoter in VSMC proliferation. VSMCs were treated with additional 5 U/ml, 10 U/ml, and 20 U/ml concentrations of Hiru after they were cocultured with conditioned medium from M1-polarized macrophages. (a) CCK-8 assay was to evaluate cell viability. ELISA assay was to measure the contents of (b) TNF-*α*, (c) IL-6, and (d) IL-10. (e) Western blot was to analyze the protein levels of Bax, Bcl-2, cleaved caspase-3, caspase-3, MMP2, iNOS, and *α*-SMA. ^∗∗^*P* < 0.01 and ^∗∗∗^*P* < 0.001 vs. control. ^#^*P* < 0.05, ^##^*P* < 0.01, and ^###^*P* < 0.001 vs. M1 medium.

After VSMCs were cultured in the conditioned medium from M1 polarized macrophages, they were treated with additional 5, 10, and 20 U/ml concentrations of Hiru. Subsequently, it turned out that the viability of VSMCs was reduced by the addition of M1-CM, which was constantly restored by increasing concentrations of Hiru (Figure 4(a)). Further, Hiru also cut down the levels of TNF-*α*, IL-6, and enhanced IL-10 content induced by M1-CM, indicating that Hiru reduced inflammatory response in M1 macrophages (Figures 4(b)–4(d)). Similarly, Hiru protected VSMCs against macrophage towards M1 activation and VSMC apoptosis and promoted their proliferation in a macrophage-VSMC coculture system, as evidenced by the downregulated Bax, cleaved caspase-3/caspase-3, MMP2, and iNOS protein levels, as well as the upregulated Bcl-2 and *α*-SMA protein levels (Figure 4(e)). To be concluded, Hiru impeded M1 macrophage-mediated inflammation and VSMC apoptosis while promoted VSMC proliferation.

### 3.5. Hiru Is Involved in VSMC Proliferation, Apoptosis, and Inflammation through Modulating M1/M2 Polarization

**Figure 5:**
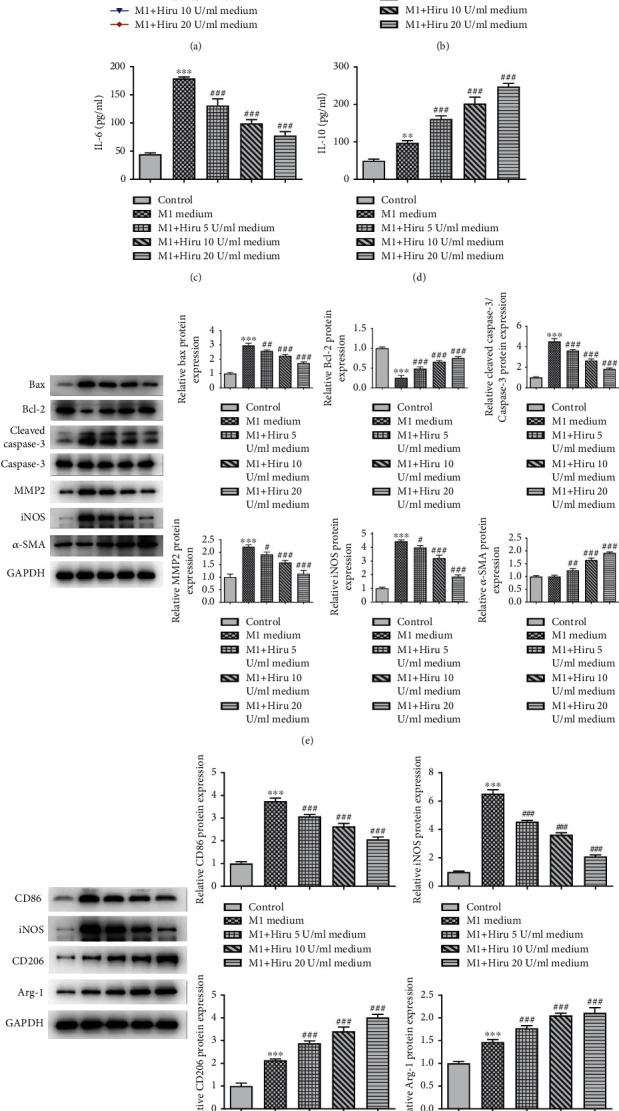
Hiru is involved in VSMC proliferation, apoptosis, and inflammation through modulating M1/M2 polarization. VSMCs were incubated with M1-CM whose cultivation process was intervened by 5 U/ml, 10 U/ml, and 20 U/ml concentrations of Hiru, respectively. (a) CCK-8 assay was to evaluate cell viability. ELISA assay was to measure the contents of (b) TNF-*α*, (c) IL-6, and (d) IL-10. (e) Western blot was to analyze the protein levels of Bax, Bcl-2, cleaved caspase-3, caspase-3, MMP2, iNOS, and *α*-SMA. (f) Western blot was to analyze CD86, iNOS, CD206, and Arg-1 protein levels in M1 macrophage. ^∗∗^*P* < 0.01 and ^∗∗∗^*P* < 0.001 vs. control. ^#^*P* < 0.05, ^##^*P* < 0.01, and ^###^*P* < 0.001 vs. M1 medium.

As displayed in Figure 5(a), the viability of VSMCs was slightly weakened by M1 medium while this result was countervailed by increasing concentrations of M1+Hiru medium to some extent. Conversely, the inflammatory response was ameliorated when the induction process of M1-CM was stimulated with different concentrations of Hiru, as evidenced by the decreased TNF-*α* and IL-6 levels, as well as increased IL-10 level in the M1+Hiru medium groups (5, 10, and 20 U/ml) relative to the M1 medium group (Figures 5(b) and 5(d)). Additionally, the addition of 5, 10, and 20 U/ml of Hiru in the cultivation process of M1 medium lessened the expressions of Bax, cleaved caspase-3/caspase-3, MMP2, and iNOS, whereas raised the expressions of Bcl-2 and *α*-SMA (Figure 5(e)). As shown in Figure 5(f), in comparison with the M1 medium group, CD86 and iNOS expressions were decreased while CD206 and Arg-1 expressions were increased in the M1+Hiru medium groups dependent on increasing concentrations of Hiru. Taken together, Hiru contributed to VSMC proliferation and impeded apoptosis and inflammation via mediating M1/M2 polarization.

## 4. Discussion

AVF is deemed as an optimal vascular access for the process of HD, which is also a widely accepted renal replacement therapy for CRF on account of its superior patency, low risk of infection, few complications, and better outcomes [23, 24]. Nevertheless, major risk factors including vascular cell proliferation and migration, extracellular matrix remodeling, complicated interactions of growth factors, adhesion molecules, inflammatory mediators, and chemokines may result in AVF maturation failure, which is likely to further improve the morbidity and hospitalization of HD patients [25, 26]. Inflammation is a protective response that eliminates the initial cause of cell injury, removes the damaged necrotic tissues and cells, and triggers tissue repair under normal conditions [27]. However, the excessive and persistent inflammatory response may contribute to deleterious autoimmune process, which might even be the initiation of human diseases in most cases [28]. As an example, the chronic inflammatory response is regarded as a representative comorbidity of CKD, especially in dialysis patients [29]. Moreover, maladaptive, uncontrolled, and continuous inflammation may aggravate CRF via interacting with factors that emerge during the accumulation process of uremic toxins [30]. More importantly, inflammation is an indispensable driver of AVF maturation failure through mediating vascular response [31]. As we know, IL-6 and TNF-*α* are proinflammatory mediators while IL-10 is a typical anti-inflammatory cytokine [32, 33]. IL-6 and TNF-*α* have been determined to display high expression in CKD while the abnormal expression of IL-10 is also implicated as a risk factor for kidney disease [34, 35].

Macrophages present distinct phenotypes: M1 macrophages and M2 macrophages are constantly changing under different environments [36]. In detail, M1 macrophages initiate and maintain inflammatory responses through secreting proinflammatory cytokines, while M2 macrophages alleviate inflammation through releasing anti-inflammatory mediators [37]. CD86 and iNOS are regarded as M1 markers, while CD206 and Arg-1 are determined as M2 markers [38, 39]. The uncontrolled production of inflammatory mediators is stimulated by altering the M1/M2 polarization of macrophages, which ultimately leads to macrophage dysfunction [40]. Accumulating evidence has confirmed that macrophages take advantage of the regulation of inflammatory response to influence renal injury and fibrosis [41]. Moreover, TGF-*β* and Arg-1 secreted by M2 macrophages may also help vascular proliferation and AVF maturation [42]. It is well documented that the polarization of proinflammatory M1 macrophages produces proinflammatory cytokines such as IL-6 and TNF-*α* after stimulation by LPS or Th1 cytokine, IFN-*γ* [43]. Hence, this study employed LPS and IFN-*γ* to induce M1 polarization in RAW264.7 cells. The experimental results elucidated that M1 macrophage polarization reduced M2 macrophage polarization and aggravated inflammatory response, as evidenced by the elevated iNOS level, M1/M2 ratio, TNF-*α*, IL-6, and IL-10 levels, as well as the decreased Arg-1 protein level in the presence of IFN-*γ* and LPS. Furthermore, the coincubation of conditioned medium from M1-polarized macrophages with VSMCs distinctly abrogated cell viability while reinforced cell apoptosis and inflammation.

Hiru is a direct inhibitor of thrombin, and its role in human diseases has been highlighted by emerging reports [13]. For instance, Yu et al. have proposed that Hiru mitigates Ang II-stimulated myocardial fibroblast fibrosis through inactivating the ERK1/2 pathway. Xie et al. have proved that Hiru protects against renal tubule injury and inflammation in UUO mice [14]. Han et al. have uncovered that Hiru attenuated kidney damage by suppressing inflammation in diabetic nephropathy [15]. Furthermore, Li et al. have illuminated that paclitaxel combined with Hiru eases inflammatory reactions of human coronary artery smooth muscle cells [16]. What is more, Hiru partially reverses the downregulated Arg-1 expression in streptozotocin-induced diabetes [17]. Consistent with these findings, after the establishment of CRF rat models, Hiru was discovered to decrease the levels of renal injury markers BUN and Scr in a concentration-dependent manner, implying that Hiru relieved renal and vascular endothelial injury while boosted vascular smooth muscle hyperplasia in CRF rat models. Meanwhile, the detection of M1 markers (CD86 and iNOS) and M2 markers (CD206 and Arg-1) revealed that Hiru facilitated the differentiation and activation of M1 macrophage towards M2 macrophage in CRF rat models. Subsequently, different concentrations of Hiru were utilized to treat VSMCs after they were cocultured with conditioned medium from M1-polarized macrophages. It turned out that Hiru motivated the viability while abrogated the apoptosis and inflammation of VSMCs in M1-CM. This result was slightly different from the one obtained by adding Hiru during the incubation of VSMCs in M1-CM. The experimental results validated that VSMCs in M1 medium suffered less injury in this condition. Also, it was apparently seen that 5, 10, and 20 U/ml concentrations of Hiru similarly motivated the viability and M2 polarization while abrogated the apoptosis, inflammation, and M1 polarization of VSMCs. These results revealed that Hiru could not only inhibit inflammation and apoptosis by a direct treatment of VSMCs but also indirectly affect VSMC damage by changing the levels of inflammatory cytokines in macrophage-conditioned medium.

## 5. Conclusions

To be concluded, Hiru exerted the protective role against vascular injury in CRF directly or via mediating the inflammation in M1/M2 macrophage polarization. Anyway, this finding might refer to Hiru as a valuable target for the AVF maturation to improve the survival rate of CRF patients.

## Data Availability

The experimental data will be available from the corresponding author on reasonable request.
